# Supplementary material for: Optimal minimal residual disease threshold in pediatric acute myeloid leukemia: A retrospective cohort study based on the TARGET database
Source: PLoS Med. 2026 May 8;23(5):e1005088. doi: 10.1371/journal.pmed.1005088 (PMC13155632; doi:10.1371/journal.pmed.1005088)
Supplement: S1 Code — (ZIP) [file pmed.1005088.s002.zip › S2 code/PROJ8_15_tbl1/PROJ8_15_tbl1.htm]

## Kaplan Meier Survival Curve

Outcome: First Event
Time: EFS(years)
Landmark analysis for time segment: EFS(years) <= 5
Log rank test: implements the G-rho family of Harrington and Fleming (1982), with weights on each death of S(t)^rho, where S is the Kaplan-Meier estimate of survival. With rho = 0 this is the log-rank or Mantel-Haenszel test.

|  |  |  |  |
| --- | --- | --- | --- |
|  | N | Observed | Expected |
| factor(X25.CS)=0 | 92 | 41 | 61.7092 |
| factor(X25.CS)=1 | 36 | 32 | 11.2908 |

Chisq=
46.6153
on
1
degree of freedom, p=
<0.0001
Survival table

|  |  |  |  |  |  |  |  |
| --- | --- | --- | --- | --- | --- | --- | --- |
|  | EFS(years) | N.Risk | N.Event | N.Censor | Survival | 95%CI Low | 95%CI Upp |
| factor(X25.CS)=0 | 0.19 | 92 | 1 | 0 | 0.9891 | 0.9682 | 1.0000 |
| factor(X25.CS)=0 | 0.21 | 91 | 2 | 0 | 0.9674 | 0.9318 | 1.0000 |
| factor(X25.CS)=0 | 0.22 | 89 | 2 | 0 | 0.9457 | 0.9004 | 0.9931 |
| factor(X25.CS)=0 | 0.32 | 87 | 1 | 0 | 0.9348 | 0.8857 | 0.9866 |
| factor(X25.CS)=0 | 0.33 | 86 | 1 | 0 | 0.9239 | 0.8713 | 0.9797 |
| factor(X25.CS)=0 | 0.41 | 85 | 1 | 0 | 0.9130 | 0.8572 | 0.9725 |
| factor(X25.CS)=0 | 0.42 | 84 | 1 | 0 | 0.9022 | 0.8435 | 0.9650 |
| factor(X25.CS)=0 | 0.44 | 83 | 2 | 0 | 0.8804 | 0.8166 | 0.9493 |
| factor(X25.CS)=0 | 0.45 | 81 | 1 | 0 | 0.8696 | 0.8034 | 0.9412 |
| factor(X25.CS)=0 | 0.53 | 80 | 1 | 0 | 0.8587 | 0.7904 | 0.9329 |
| factor(X25.CS)=0 | 0.55 | 79 | 2 | 0 | 0.8370 | 0.7648 | 0.9159 |
| factor(X25.CS)=0 | 0.66 | 77 | 1 | 0 | 0.8261 | 0.7522 | 0.9073 |
| factor(X25.CS)=0 | 0.67 | 76 | 1 | 0 | 0.8152 | 0.7396 | 0.8985 |
| factor(X25.CS)=0 | 0.74 | 75 | 1 | 0 | 0.8043 | 0.7272 | 0.8896 |
| factor(X25.CS)=0 | 0.76 | 74 | 1 | 0 | 0.7935 | 0.7149 | 0.8807 |
| factor(X25.CS)=0 | 0.81 | 73 | 1 | 0 | 0.7826 | 0.7027 | 0.8716 |
| factor(X25.CS)=0 | 0.84 | 72 | 5 | 0 | 0.7283 | 0.6428 | 0.8251 |
| factor(X25.CS)=0 | 0.85 | 67 | 1 | 0 | 0.7174 | 0.6310 | 0.8156 |
| factor(X25.CS)=0 | 0.91 | 66 | 3 | 0 | 0.6848 | 0.5961 | 0.7866 |
| factor(X25.CS)=0 | 1.05 | 63 | 1 | 0 | 0.6739 | 0.5846 | 0.7768 |
| factor(X25.CS)=0 | 1.09 | 62 | 1 | 0 | 0.6630 | 0.5732 | 0.7670 |
| factor(X25.CS)=0 | 1.11 | 61 | 0 | 2 | 0.6630 | 0.5732 | 0.7670 |
| factor(X25.CS)=0 | 1.15 | 59 | 0 | 1 | 0.6630 | 0.5732 | 0.7670 |
| factor(X25.CS)=0 | 1.16 | 58 | 1 | 0 | 0.6516 | 0.5611 | 0.7568 |
| factor(X25.CS)=0 | 1.17 | 57 | 1 | 0 | 0.6402 | 0.5490 | 0.7464 |
| factor(X25.CS)=0 | 1.18 | 56 | 1 | 0 | 0.6287 | 0.5371 | 0.7361 |
| factor(X25.CS)=0 | 1.25 | 55 | 0 | 1 | 0.6287 | 0.5371 | 0.7361 |
| factor(X25.CS)=0 | 1.37 | 54 | 0 | 1 | 0.6287 | 0.5371 | 0.7361 |
| factor(X25.CS)=0 | 1.39 | 53 | 2 | 1 | 0.6050 | 0.5123 | 0.7145 |
| factor(X25.CS)=0 | 1.45 | 50 | 1 | 0 | 0.5929 | 0.4997 | 0.7035 |
| factor(X25.CS)=0 | 1.55 | 49 | 2 | 0 | 0.5687 | 0.4748 | 0.6812 |
| factor(X25.CS)=0 | 1.61 | 47 | 0 | 1 | 0.5687 | 0.4748 | 0.6812 |
| factor(X25.CS)=0 | 1.83 | 46 | 0 | 1 | 0.5687 | 0.4748 | 0.6812 |
| factor(X25.CS)=0 | 2.14 | 45 | 1 | 0 | 0.5561 | 0.4618 | 0.6696 |
| factor(X25.CS)=0 | 2.37 | 44 | 1 | 0 | 0.5434 | 0.4489 | 0.6579 |
| factor(X25.CS)=0 | 2.46 | 43 | 0 | 1 | 0.5434 | 0.4489 | 0.6579 |
| factor(X25.CS)=0 | 2.73 | 42 | 0 | 1 | 0.5434 | 0.4489 | 0.6579 |
| factor(X25.CS)=0 | 3.04 | 41 | 0 | 2 | 0.5434 | 0.4489 | 0.6579 |
| factor(X25.CS)=0 | 3.28 | 39 | 0 | 1 | 0.5434 | 0.4489 | 0.6579 |
| factor(X25.CS)=0 | 3.48 | 38 | 0 | 1 | 0.5434 | 0.4489 | 0.6579 |
| factor(X25.CS)=0 | 3.74 | 37 | 0 | 1 | 0.5434 | 0.4489 | 0.6579 |
| factor(X25.CS)=0 | 3.84 | 36 | 0 | 1 | 0.5434 | 0.4489 | 0.6579 |
| factor(X25.CS)=0 | 3.87 | 35 | 0 | 1 | 0.5434 | 0.4489 | 0.6579 |
| factor(X25.CS)=0 | 3.89 | 34 | 0 | 1 | 0.5434 | 0.4489 | 0.6579 |
| factor(X25.CS)=0 | 3.9 | 33 | 0 | 1 | 0.5434 | 0.4489 | 0.6579 |
| factor(X25.CS)=0 | 4.05 | 32 | 0 | 1 | 0.5434 | 0.4489 | 0.6579 |
| factor(X25.CS)=0 | 4.33 | 31 | 0 | 1 | 0.5434 | 0.4489 | 0.6579 |
| factor(X25.CS)=0 | 4.6 | 30 | 0 | 1 | 0.5434 | 0.4489 | 0.6579 |
| factor(X25.CS)=0 | 4.73 | 29 | 0 | 1 | 0.5434 | 0.4489 | 0.6579 |
| factor(X25.CS)=0 | 5 | 28 | 0 | 28 | 0.5434 | 0.4489 | 0.6579 |
| factor(X25.CS)=1 | 0.13 | 36 | 2 | 0 | 0.9444 | 0.8725 | 1.0000 |
| factor(X25.CS)=1 | 0.15 | 34 | 1 | 0 | 0.9167 | 0.8307 | 1.0000 |
| factor(X25.CS)=1 | 0.16 | 33 | 2 | 0 | 0.8611 | 0.7552 | 0.9818 |
| factor(X25.CS)=1 | 0.17 | 31 | 2 | 0 | 0.8056 | 0.6861 | 0.9458 |
| factor(X25.CS)=1 | 0.18 | 29 | 2 | 0 | 0.7500 | 0.6211 | 0.9057 |
| factor(X25.CS)=1 | 0.19 | 27 | 2 | 0 | 0.6944 | 0.5592 | 0.8625 |
| factor(X25.CS)=1 | 0.2 | 25 | 1 | 0 | 0.6667 | 0.5292 | 0.8399 |
| factor(X25.CS)=1 | 0.21 | 24 | 1 | 0 | 0.6389 | 0.4998 | 0.8167 |
| factor(X25.CS)=1 | 0.22 | 23 | 2 | 0 | 0.5833 | 0.4426 | 0.7688 |
| factor(X25.CS)=1 | 0.24 | 21 | 1 | 0 | 0.5556 | 0.4148 | 0.7441 |
| factor(X25.CS)=1 | 0.25 | 20 | 1 | 0 | 0.5278 | 0.3875 | 0.7189 |
| factor(X25.CS)=1 | 0.28 | 19 | 2 | 0 | 0.4722 | 0.3343 | 0.6670 |
| factor(X25.CS)=1 | 0.29 | 17 | 1 | 0 | 0.4444 | 0.3085 | 0.6404 |
| factor(X25.CS)=1 | 0.34 | 16 | 1 | 0 | 0.4167 | 0.2831 | 0.6133 |
| factor(X25.CS)=1 | 0.49 | 15 | 1 | 0 | 0.3889 | 0.2582 | 0.5857 |
| factor(X25.CS)=1 | 0.5 | 14 | 1 | 0 | 0.3611 | 0.2339 | 0.5576 |
| factor(X25.CS)=1 | 0.62 | 13 | 1 | 0 | 0.3333 | 0.2100 | 0.5291 |
| factor(X25.CS)=1 | 0.64 | 12 | 2 | 0 | 0.2778 | 0.1640 | 0.4704 |
| factor(X25.CS)=1 | 0.72 | 10 | 1 | 0 | 0.2500 | 0.1420 | 0.4402 |
| factor(X25.CS)=1 | 0.91 | 9 | 1 | 0 | 0.2222 | 0.1206 | 0.4094 |
| factor(X25.CS)=1 | 1.07 | 8 | 1 | 0 | 0.1944 | 0.1000 | 0.3781 |
| factor(X25.CS)=1 | 1.15 | 7 | 2 | 0 | 0.1389 | 0.0616 | 0.3133 |
| factor(X25.CS)=1 | 1.74 | 5 | 1 | 0 | 0.1111 | 0.0441 | 0.2799 |
| factor(X25.CS)=1 | 5 | 4 | 0 | 4 | 0.1111 | 0.0441 | 0.2799 |

|  |  |  |  |  |  |  |  |  |  |
| --- | --- | --- | --- | --- | --- | --- | --- | --- | --- |
| X25.CS | records | n.max | n.start | events | \*rmean | \*se(rmean) | median | 0.95LCL | 0.95UCL |
| factor(X25.CS)=0 | 92 | 92 | 92 | 41 | 3.109 | 0.221 | NA | 1.45 | NA |
| factor(X25.CS)=1 | 36 | 36 | 36 | 32 | 0.944 | 0.247 | 0.28 | 0.21 | 0.64 |

Landmark analysis for time segment: EFS(years) > 5
Log rank test: implements the G-rho family of Harrington and Fleming (1982), with weights on each death of S(t)^rho, where S is the Kaplan-Meier estimate of survival. With rho = 0 this is the log-rank or Mantel-Haenszel test.

|  |  |  |  |
| --- | --- | --- | --- |
|  | N | Observed | Expected |
| factor(X25.CS)=0 | 28 | 4 | 3.3000 |
| factor(X25.CS)=1 | 4 | 0 | 0.7000 |

Chisq=
0.9287
on
1
degree of freedom, p=
0.3352
Survival table

|  |  |  |  |  |  |  |  |
| --- | --- | --- | --- | --- | --- | --- | --- |
|  | EFS(years) | N.Risk | N.Event | N.Censor | Survival | 95%CI Low | 95%CI Upp |
| factor(X25.CS)=0 | 5.05 | 28 | 0 | 1 | 1.0000 | 1.0000 | 1.0000 |
| factor(X25.CS)=0 | 5.14 | 27 | 0 | 1 | 1.0000 | 1.0000 | 1.0000 |
| factor(X25.CS)=0 | 5.32 | 26 | 0 | 1 | 1.0000 | 1.0000 | 1.0000 |
| factor(X25.CS)=0 | 5.35 | 25 | 0 | 1 | 1.0000 | 1.0000 | 1.0000 |
| factor(X25.CS)=0 | 5.55 | 24 | 0 | 1 | 1.0000 | 1.0000 | 1.0000 |
| factor(X25.CS)=0 | 5.7 | 23 | 0 | 1 | 1.0000 | 1.0000 | 1.0000 |
| factor(X25.CS)=0 | 5.74 | 22 | 0 | 1 | 1.0000 | 1.0000 | 1.0000 |
| factor(X25.CS)=0 | 5.8 | 21 | 0 | 1 | 1.0000 | 1.0000 | 1.0000 |
| factor(X25.CS)=0 | 6 | 20 | 0 | 1 | 1.0000 | 1.0000 | 1.0000 |
| factor(X25.CS)=0 | 6.04 | 19 | 0 | 1 | 1.0000 | 1.0000 | 1.0000 |
| factor(X25.CS)=0 | 6.15 | 18 | 0 | 1 | 1.0000 | 1.0000 | 1.0000 |
| factor(X25.CS)=0 | 6.39 | 17 | 0 | 1 | 1.0000 | 1.0000 | 1.0000 |
| factor(X25.CS)=0 | 6.4 | 16 | 0 | 1 | 1.0000 | 1.0000 | 1.0000 |
| factor(X25.CS)=0 | 6.45 | 15 | 3 | 0 | 0.8000 | 0.6212 | 1.0000 |
| factor(X25.CS)=0 | 6.56 | 12 | 1 | 0 | 0.7333 | 0.5405 | 0.9950 |
| factor(X25.CS)=0 | 6.59 | 11 | 0 | 1 | 0.7333 | 0.5405 | 0.9950 |
| factor(X25.CS)=0 | 6.65 | 10 | 0 | 2 | 0.7333 | 0.5405 | 0.9950 |
| factor(X25.CS)=0 | 6.75 | 8 | 0 | 1 | 0.7333 | 0.5405 | 0.9950 |
| factor(X25.CS)=0 | 6.79 | 7 | 0 | 1 | 0.7333 | 0.5405 | 0.9950 |
| factor(X25.CS)=0 | 6.89 | 6 | 0 | 1 | 0.7333 | 0.5405 | 0.9950 |
| factor(X25.CS)=0 | 6.92 | 5 | 0 | 1 | 0.7333 | 0.5405 | 0.9950 |
| factor(X25.CS)=0 | 6.94 | 4 | 0 | 1 | 0.7333 | 0.5405 | 0.9950 |
| factor(X25.CS)=0 | 7.36 | 3 | 0 | 1 | 0.7333 | 0.5405 | 0.9950 |
| factor(X25.CS)=0 | 7.53 | 2 | 0 | 1 | 0.7333 | 0.5405 | 0.9950 |
| factor(X25.CS)=0 | 8.28 | 1 | 0 | 1 | 0.7333 | 0.5405 | 0.9950 |
| factor(X25.CS)=1 | 6.19 | 4 | 0 | 1 | 1.0000 | 1.0000 | 1.0000 |
| factor(X25.CS)=1 | 7 | 3 | 0 | 2 | 1.0000 | 1.0000 | 1.0000 |
| factor(X25.CS)=1 | 8.15 | 1 | 0 | 1 | 1.0000 | 1.0000 | 1.0000 |

|  |  |  |  |  |  |  |  |  |  |
| --- | --- | --- | --- | --- | --- | --- | --- | --- | --- |
| X25.CS | records | n.max | n.start | events | \*rmean | \*se(rmean) | median | 0.95LCL | 0.95UCL |
| factor(X25.CS)=0 | 28 | 28 | 28 | 4 | 7.752 | 0.198 | NA | NA | NA |
| factor(X25.CS)=1 | 4 | 4 | 4 | 0 | 8.215 | 0 | NA | NA | NA |

Use subset of data: (!is.na(X5) & (X5==3))
Created by EmpowerStats (www.empowerstats.com) and R on 2025-10-07
